# Disentangling the genetic bases of *Saccharomyces cerevisiae* nitrogen consumption and adaptation to low nitrogen environments in wine fermentation

**DOI:** 10.1186/s40659-019-0270-3

**Published:** 2020-01-09

**Authors:** Eduardo I. Kessi-Pérez, Jennifer Molinet, Claudio Martínez

**Affiliations:** 1 Departamento de Ciencia y Tecnología de los Alimentos, Universidad de Santiago de Chile (USACH), Santiago, Chile; 2 Centro de Estudios en Ciencia y Tecnología de Alimentos (CECTA), Universidad de Santiago de Chile (USACH), Santiago, Chile

**Keywords:** *Saccharomyces cerevisiae*, Wine yeasts, Nitrogen consumption, Wine fermentation, Natural variation, Wild alleles, QTL mapping, GWAS, ASE, TORC1 pathway

## Abstract

The budding yeast *Saccharomyces cerevisiae* has been considered for more than 20 years as a premier model organism for biological sciences, also being the main microorganism used in wide industrial applications, like alcoholic fermentation in the winemaking process. Grape juice is a challenging environment for *S. cerevisiae*, with nitrogen deficiencies impairing fermentation rate and yeast biomass production, causing stuck or sluggish fermentations, thus generating sizeable economic losses for wine industry. In the present review, we summarize some recent efforts in the search of causative genes that account for yeast adaptation to low nitrogen environments, specially focused in wine fermentation conditions. We start presenting a brief perspective of yeast nitrogen utilization under wine fermentative conditions, highlighting yeast preference for some nitrogen sources above others. Then, we give an outlook of *S. cerevisiae* genetic diversity studies, paying special attention to efforts in genome sequencing for population structure determination and presenting QTL mapping as a powerful tool for phenotype–genotype correlations. Finally, we do a recapitulation of *S. cerevisiae* natural diversity related to low nitrogen adaptation, specially showing how different studies have left in evidence the central role of the TORC1 signalling pathway in nitrogen utilization and positioned wild *S. cerevisiae* strains as a reservoir of beneficial alleles with potential industrial applications (e.g. improvement of industrial yeasts for wine production). More studies focused in disentangling the genetic bases of *S. cerevisiae* adaptation in wine fermentation will be key to determine the domestication effects over low nitrogen adaptation, as well as to definitely proof that wild *S. cerevisiae* strains have potential genetic determinants for better adaptation to low nitrogen conditions.

## Background

The budding yeast *Saccharomyces cerevisiae* (hereinafter, called “*S. cerevisiae*” or simply “yeast”) has been considered for more than 20 years as one of the main model organisms for biological sciences such as genetics and molecular biology, being the first eukaryotic organism with its genome fully sequenced in 1996, milestone that required a combined international effort [1]. Nowadays, yeast continues being a workhorse to assess different biological questions, such as the minimal genome needed for cell functionality and, probably, it will be soon the first synthetic eukaryotic genome [2, 3].

Beyond of its extraordinary facility for genetic manipulation and the wide repertory of molecular biology techniques for genome edition, *S. cerevisiae* is also the main microorganism industrially utilized worldwide and a biological platform for biotechnology, with applications that included production of wine, beer, bread, heterologous proteins, vaccines and high value metabolites [4–8]. Altogether, the uses of yeast as model organism and its multiple biotechnological applications have boosted the genome sequencing of a great number of strains, revelling the genomics features that permit its adaptation to diverse ecological niches, including industrial ones, as is the case with wine fermentation [9, 10].

*Saccharomyces cerevisiae* is the main microorganism responsible for the alcoholic fermentation in the winemaking process, contributing not only with the alcoholic degree but also with flavours and aromas to the final product [11, 12]. In this context, this species has different adaptations to the fermentative environments; a very interesting recent review discusses the mechanisms of yeast adaptation to wine fermentation from an “omics” point of view [13]. For example, *S. cerevisiae* is a Crabtree positive yeast, i.e. in the presence of a fermentable carbon source (like glucose or fructose), its metabolism proceeds mainly via fermentation rather than respiration, even in the presence of oxygen [14]. This Crabtree positive condition is ecologically important for *S. cerevisiae* because wine fermentation is a complex microbiological process, where the rapid transformation of sugars (glucose and fructose) into ethanol allows it to outcompete other present microorganisms [15] (for new perspectives in the subject, see [16, 17]).

Furthermore, the grape juice itself is a challenging environment for yeasts, where is necessary face up to temperature fluctuations, low O_2_, low pH (between 2 and 3), high osmotic pressure (20% of sugar concentration), high sulphite levels, ethanol toxicity and nutritional deficiencies [13, 18, 19]. For example, at the beginning of wine fermentation a strong osmotic stress is induced by high sugar concentrations, potentially causing a reduction of both growth and viability, which is sensed by yeast cells through multiple signalling pathways that allow them to quickly respond to altered osmolarity [20]. In contrast to sugar, *S. cerevisiae* has to adapt to the depletion of essential nutrients during first stages of fermentation; in this context, nitrogen is the main limiting nutrient during wine fermentation, being highly correlated with fermentation kinetics [8, 21].

Nitrogen deficiencies in grape juice impairs the fermentation rate and yeast biomass production, which causes stuck or sluggish fermentations [21], which is recognized as one of the main problems in wine industry, generating sizeable economic losses [22]. In addition, nitrogen deficiencies mainly affect the highest quality red wines, due to the complex composition of the grape must utilized in its production, which contains tannins and phenols that induce the stress response in yeast [23, 24]. In this context, winemakers try to solve this problem by two main strategies: (i) vineyard management through the use of nitrogen fertilizers and (ii) nitrogen supplementation of the grape must [25]. This suggests that industrial wine yeasts are not well adapted to low nitrogen environments, requiring high levels of nitrogen to complete the fermentation process [26].

In the present review, we summarize some recent efforts in the search of causative genes that account for yeast adaptation to low nitrogen environments, specially focused in wine fermentation conditions. To achieve this, we start by presenting a brief perspective of yeast nitrogen utilization under wine fermentative conditions, then an outlook of yeast genetic diversity studies, and finally a recapitulation of yeast natural diversity related to low nitrogen adaptation, with special emphasis in the central role of the TORC1 signalling pathway in nitrogen utilization and the idea that wild *S. cerevisiae* strains are a reservoir of beneficial alleles with potential industrial applications (due to its impact in processes related to nitrogen sensing and uptake).

## Main text

### Yeast nitrogen utilization under wine fermentative conditions

Several reviews exist that focus on yeast nitrogen sensing, signalling, transport, assimilation and metabolism [27–35]. A general conclusion is that yeast growth rate is not only associated with the amount of available nitrogen, but also with the quality of the nitrogen source [18, 27]. Thus, nitrogen sources sustaining high growth rate such as glutamine, glutamate, asparagine and ammonium are considered as preferred, whereas proline, allantoin and urea allows slow growth rate and, therefore, are considered as non-preferred nitrogen sources [28].

Nitrogen can be found in various forms in grape must, with yeast consuming mainly ammonium and amino acids; from them, the main amino acids present are proline, arginine, alanine, glutamate, glutamine, serine and threonine, in addition to ammonia [36]. Moreover, in wine fermentation context these nitrogen sources are consumed following a specific order, where asparagine, threonine, glutamine, leucine, histidine, methionine, isoleucine, serine, glycine and phenylalanine are utilized at the beginning of the fermentation, while ammonium, valine, arginine, alanine, tryptophan and tyrosine are preferred at later times [37]. This preference for different nitrogen sources is the aftermath of a tight metabolic regulation system where four different mechanisms regulate nitrogen utilization: Ssy1-Ptr3-Ssy5 system (SPS), nitrogen catabolic repression (NCR), retrograde signalling pathway (RTG) and the general control of amino acids (GAAC); with all of them in turn regulated by the TORC1 signalling pathway [29, 30].

Under nitrogen limited conditions, yeast cells grow slowly, reducing the ribosome biogenesis, protein translation and arresting the cell cycle in G1 [38]. There are a couple of excellent reviews covering this topic in wine fermentation context [18, 21, 26], mainly focused on studies that used media containing a mixture of different nitrogen sources mimicking natural grape musts. Remarkably, several groups around the world have shown that the nitrogen requirements of *S. cerevisiae* are strain-dependent, i.e. different yeast strains have different necessities of nitrogen, and then have different capacities to growth and perform wine fermentation in nitrogen-limited musts [39–43]. In this context, the wide phenotypic diversity observed in *S. cerevisiae* for nitrogen requirements is probably a consequence of its genetic diversity, being an important challenge to disentangle this diversity.

### Yeast genetic diversity

#### Genome sequencing and population structure

As abovementioned, *S. cerevisiae* is a model organism with its genome fully sequenced since 1996 [1]. Since then, different attempts have been made to unveil the genetic diversity and population structure of the species. The first attempts to unveil the genetic diversity present in *S. cerevisiae* were done sequencing individual genes and using molecular markers, which showed the presence of two main yeast populations: domesticated yeasts associated with human activities (wine, beer, bread, etc.) and wild yeasts from natural environments without human intervention [44–46].

These two populations reflect different evolutionary trajectories, for instance, wine yeasts have been selected by centuries of human activity, preferring traits such as ethanol production and fruity flavours and fragrances; while wild yeasts have faced challenging environments with scarcity of carbon and nitrogen sources [47]. Furthermore, this divergent track of selection was confirmed by assaying the ability of wild yeasts to growth in a wide range of carbon and nitrogen sources, which contrasts with the limited nitrogen and carbon sources sustaining growth in wine yeasts [48].

After these first attempts, the population structure of *S. cerevisiae* was finally resolved by genome sequencing of 37 yeast strains isolated from different ecological niches, demonstrating the presence of five clean lineages or subpopulations in the species: Malaysian (MA), Sake (SA), North American (NA), West African (WA) and Wine/European (WE) [49, 50]. Afterward, the genome sequencing of 100 yeast strains confirmed the presence of these five clean lineages (MA, NA, SA, WA and WE) in the population structure of *S. cerevisiae* [51]. Interestingly, the information obtained by these and other sequencing efforts has revealed unique sets of genetic features related with yeast strains specific niche adaptation, often absent in the reference genome, some of them acquired by horizontal gene transfer events from distant species or by introgression from closely relative ones [52–58].

Recently, the “1002 yeast genomes project” has been finished, representing the most complete catalogue of the genetic variation in *S. cerevisiae*, where a population of 1011 yeast strains isolated from different ecological niches has been sequenced [59]. In the “1011 population”, a total of 26 clades were described, expanding the number of phylogenetic clusters initially observed in the species; of them, 362 isolates were grouped into the WE cluster [59]. Additionally, the sequencing of this huge population confirmed that isolates from Asia have the greatest genetic diversity within the species [59, 60]. Nowadays, the repertory of yeast strains with sequenced genomes includes wild isolates from environments such as tree bark and flowers, and domesticated isolates from clinical, dairy, cheese, brewery and vineyard environments (among others) [49, 51, 54, 59–62]; with the “1011 yeasts population” including most the genetic variation existing in *S. cerevisiae* and becoming a powerful resource for genotype–phenotype correlations [59].

#### Phenotype–genotype correlation by QTL mapping

The extensive knowledge and information generated by the sequencing projects in *S. cerevisiae* has been accompanied by massive phenotyping efforts under different culture conditions, showing that phenotypic variation is wider than genotypic diversity in the species [63, 64]. This observation suggests that yeast has a broad phenotypic plasticity and, more importantly, that multiple genes contributes to most of the phenotypes studied, which are commonly refers as polygenic traits or complex traits [65].

QTL mapping has been the main experimental approximation to fill the gap between genotype and phenotype in yeast [66]. In this approach, a population of individuals derived from a cross is genotyped and phenotyped, allowing the statistical correlation between genotype and phenotype to map genes affecting the trait of interest [67, 68]. This approach has been extensively used in yeast for mapping causatives genes affecting phenotypes such as thermotolerance [69–71], chemical resistance [69, 72], translation termination [73] and dehydration stress tolerance [74], among many others, being particularly important in identifying quantitative trait nucleotides impacting technological performances of industrial yeast strains (see [75] for more details in the topic).

In this context, several QTLs have been mapped for phenotypes associated with the fermentation process, such as ethanol production, residual sugar and acidity [76–80], as well as QTLs involved in nitrogen-limited fermentations and in nitrogen consumption and utilization [41, 42, 78, 81–85] (Table 1). Overall, QTL mapping has proved to be an efficient tool for the detection of genes responsible of the phenotypic variation observed in populations generated by crosses, particularly for fermentative phenotypes.

**Table 1:** Examples of nitrogen-associated genes identified by QTL mapping and other approaches

Phenotype under study			Experimental condition			Method(s) of detection	Gene(s) identified	Reference(s)
A	B	C	D	E	F			
	X	X	X		X	QTL mapping (ISA) plus microarray	*ABZ1*	[76]
X			X	X	X	MHA	*ADE5,7*, *ARO8*, *VBA3*	[40]
		X	X		X	QTL mapping (BSA)	*ARG8*, *BIO3*, *GCN1*, *MDS3*	[82]
	X		X		X	QTL mapping (ISA)	*AGP1*, *ASI1*, *GLT1*	[41]
X				X		QTL mapping (ISA)	*DAL1*, *DAL4*, *RIM15*, *PUT4*	[48]
	X	X	X	X	X	QTL mapping (ISA)	*RIM15*	[42]
	X		X		X	ASE plus ASB	*ASN1*	[90]
		X	X		X	ASE	*GDB1*	[91]
	X		X		X	QTL mapping (ISA) plus BSR-seq	*ARO1*, *ALP1*, *ASI2*, *CPS1*, *EAP1*, *GTR1*, *LYP1*, *NPR1*, *PDC1*, *RPI1*, *SAP185*, *SCH9*, *SIT4*, *TOR2*	[81, 83, 85]
		X		X	X	WYDC	*AAT2*, *BRO1*, *EAR1*, *MFA2*, *MMS2*, *MRP17*, *MVB12*, *TPK2*, *UBC13*, *UBI4, UBP7*	[89]
		X	X	X	X	QTL mapping (ISA)	*KAE1*	[84]

A: Growth kinetics. B: nitrogen sources consumption. C: fermentation kinetics. D: nitrogen-sufficient condition. E: nitrogen-limited condition. F: wine condition

### Yeast natural diversity related to low nitrogen adaptation

#### Nitrogen consumption

Several genes have been linked with yeast strains differences in nitrogen consumption, under laboratory and/or fermentative conditions, not only by QTL mapping but also using other experimental strategies (Table 1). For example, transcriptomic studies have given insights into groups of genes related to nitrogen uptake [86], nitrogen requirements [39] and response to nitrogen availability [87, 88], all of them using wine strains. In other approach, a massive hemizygote analysis showed that the inability of a commercial wine strain to utilize methionine is a consequence of mutations in *ADE5,7*, *ARO8* and *VBA3* genes [40]. In a similar way, the use of a wine yeast deletion collection (WYDC) showed that deletion of *MFA2* resulted in a decrease in fermentation duration in nitrogen-limited conditions [89].

In a different genetic strategy, allele specific expression (ASE), an approach that allows the study of eQTLs (expression QTLs) through the combination of recombinant populations (as in traditional QTL mapping) and sequencing-based methods (RNA-seq), has also been used in studies that showed that coding and non-coding mutations in *ASN1* explains nitrogen consumption differences between different yeast strains [90] and that polymorphisms within the coding region of *GDB1* underlie fermentation kinetics differences [91].

Anyway, QTL mapping has been a preferred method for detection of genes linked to industrial-importance phenotypes in general (reviewed in [75]) and nitrogen consumption in particular (Table 1). One strategy has been the use of recombinant populations derived from wine strains. For example, using a recombinant population derived from two wine strains and phenotyped for nitrogen requirements for efficient fermentation, *ARG8*, *BIO3*, *GCN1* and *MDS3* genes were linked to key roles in nitrogen metabolism and signalling [82]. Other study found, utilizing a recombinant population derived from a wine and a laboratory strain, that *ABZ1* gene controls the fermentation rate through modulation of nitrogen utilization [76].

In contrast, another useful strategy is to use strains representative of the clean lineages abovementioned for *S. cerevisiae* [49] as the parental strains of the recombinant populations. Through this approach, several genes have been mapped and validated: *AGP1*, *ASI1* and *GLT1* for variation in nitrogen sources consumption underlying differences in the central nitrogen metabolism [41]; *DAL1*, *DAL4*, *RIM15* and *PUT4* for nitrogen source use variations [48]; and *RIM15*, this last gene having an antagonistic pleiotropy, with a wine strain allele conferring a greater nitrogen utilization efficiency and glycerol production but also fungicide sensitivity [42].

An alternative to the use of these bi-parental recombinant populations is the utilization of the SGRP-4X population, a multi-parental recombinant population derived from four representative strains from lineages NA, SA, WA and WE [92]. Using this population and a combined strategy of QTL mapping and BSR-seq (bulk segregant RNA-seq), allelic variants in a large set of genes (*ARO1*, *ALP1*, *ASI2*, *CPS1*, *EAP1*, *GTR1*, *LYP1*, *NPR1*, *PDC1*, *RPI1*, *SAP185*, *SCH9*, *SIT4* and *TOR2*) were identified as responsible for nitrogen consumption differences during wine fermentation [81, 83, 85]. Interestingly, half of these genes (*EAP1*, *GTR1*, *NPR1*, *SAP185*, *SCH9*, *SIT4* and *TOR2*) are directly linked to the TORC1 signalling pathway in yeast [85].

#### TORC1 pathway-associated genes

TORC1 signalling pathway is a pleiotropic signalling pathway, conserved through the eukaryotic domain from yeast to humans (where is known as “mTORC1”), that connects nutrient availability with growth, playing a central role in general metabolism regulation, specially linked to nitrogen metabolism [26]. In nitrogen starvation conditions, TORC1 activates autophagy, stress response genes, nitrogen catabolic genes and ammonium permeases; on the contrary, it represses protein biosynthesis, amino acid biosynthesis, translation initiation and ribosome biogenesis [28].

**Fig. 1:**
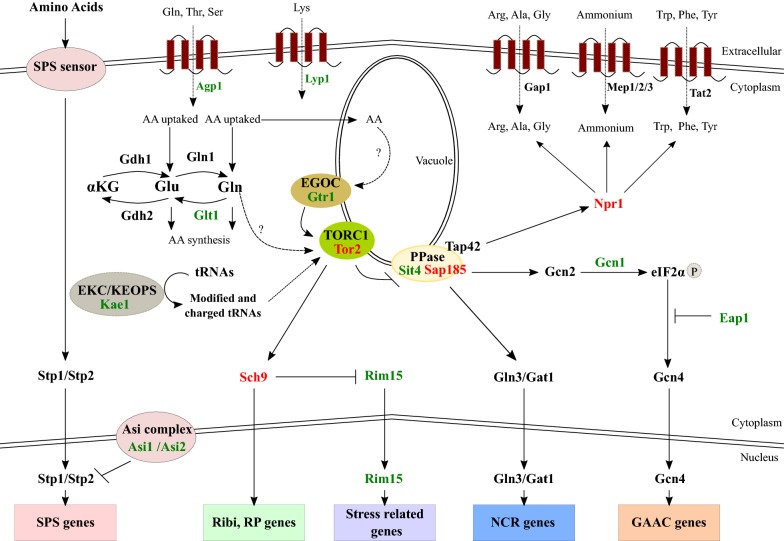
Central role of TORC1 signalling pathway and importance of wild alleles for yeast nitrogen consumption. The proteins encoded by nitrogen-associated genes identified in the studies summarized in this review are highlighted in colour (green or red). Among them, proteins encoded by genes for which wild alleles cause higher consumption levels for some nitrogen sources are highlighted in red, while the rest are in green

One of the major actual challenges in the TORC1 field is to determine exactly how intracellular levels of nitrogen are sensed by the TORC1 complex and how this pathway differentiate between preferred and non-preferred nitrogen sources [29, 30, 93, 94]. In this sense, a recently developed method allows the indirect monitoring of TORC1 activation for hundreds of strains, enabling the study of this phenotype by approaches like QTL mapping [95]. Using these experimental capacities, *KAE1* allelic variants were identified to affect both TORC1 activation by glutamine and fermentation kinetics under nitrogen-sufficient and nitrogen-limited wine conditions [84] (Fig. 1).

We can add to this last antecedent the fact that that allelic variants of *EAP1*, *GTR1*, *NPR1*, *SAP185*, *SCH9*, *SIT4* and *TOR2*, all of them genes directly associated to the yeast TORC1 pathway, underlie differences in ammonium and amino acids consumption under wine fermentation conditions [85] (Fig. 1). Also, allelic variants of *RIM15* are involved in nitrogen source use variations [47] and utilization efficiency [41], with Rim15 being downstream TORC1. Thus, the experimental evidence obtained to date reinforces the suggested idea of the importance of this pleiotropic pathway (i.e. a pathway that contribute to the regulation of multiple developmental outcomes) in yeast adaptation to low nitrogen environments in wine fermentation [26]. It this sense, it is well stablished that signalling mechanisms that control development in yeast are highly pleiotropic, implying that perturbations of signalling pathways like TORC1 can manifest in multiple and/or unexpected ways [96].

#### Wild yeast strains as reservoir of beneficial alleles

Surprisingly, for four of the aforementioned TORC1-related genes (*NPR1*, *SAP185*, *SCH9* and *TOR2*), allelic variants from the wild NA strain (which correspond to an oak tree isolate [49]) presented higher consumption levels for certain amino acids (aspartic acid, histidine, glutamine and threonine) [85] (Fig. 1). Conversely, several studies have shown that alleles coming from the domesticated WE strain (which was isolated from a winemaking environment [49]) presented lower consumption levels for particular amino acids in comparison to other strains (NA, SA and WA) [41, 81, 83, 85].

These facts are quite interestingly, because one might thought that the WE strain would be better adapted to wine fermentation conditions that, for example, the NA strain, which is a wild isolate not adapted to this environment [49]. While this is true for ammonium consumption (a nitrogen source regularly in high proportion in grape must [36]), with alleles coming from WE strain causing higher rates of it [85], it is not for the consumption of certain amino acids (as abovementioned). It is still unclear if these amino acids are present in some natural environments (like oak bark) but absent in wine environments and if this is related to the specific demand for each amino acid, information that could help to better understand the differential nitrogen consumption between wine and wild yeast strains.

A possible explanation to this phenomenon is that, while wine yeast strains have been selected by humans for phenotypes directly linked to wine final characteristics (e.g. ethanol production and fermentation kinetics properties), wild yeast strains face environments with nitrogen limitations [47], being able to growth in a wider range of nitrogen sources than wine strains [48]. Therefore, these different evolutionary trajectories between wild and wine yeasts could have led to the existence of different genetic adaptations to nitrogen-limited environments, with wild strains better adapted to this condition, thus being a reservoir of beneficial alleles from an industrial point of view.

However, an alternative explanation is that wine yeasts have high nitrogen requirements because of the production of high concentrations of ethanol, while wild yeast only need the (low) nitrogen enough to allow growth and survival. Therefore, these differences could be a consequence of their metabolism related to the substrate where they develop rather than an environmental adaptation. New evidences towards one or another explanation are needed to gain a better understanding on this topic, like genomic and/or transcriptomic studies in nitrogen-limited fermentations comparing wine and wild yeast strains.

Even so, the hypothesis that wild yeast strains are better adapted to nitrogen-limited conditions is reinforced by the fact that alleles coming from WE strain tend to cause higher rates of ammonium consumption [85], which may be caused by the regular high proportion of this nitrogen source in the grape must [36] and the oenological practice of ammonium must supplementation [25]. All these antecedents are suggesting that wine yeasts are not well adapted to low nitrogen environments, requiring high levels of nitrogen to complete the fermentation process [26]. Therefore, wild allelic variants augmenting amino acid consumption could be of industrial potential, e.g. for the improvement of industrial wine strains, maybe favouring amino acids consumption over ammonium, by genetic improvement programs.

### Future directions

Although QTL mapping and “omics”-linked approaches have been the preferred tools for the identification of genes associated with yeast adaptation to low nitrogen fermentative conditions, another experimental strategy that can also be applied for phenotype–genotype correlations is Genome-wide association study (GWAS) [66]. This approach is based on a population of individuals without direct genetic relationship, which has been genotyped (generally by chips or sequencing) and phenotyped (for a specific trait), also allowing a direct correlation between genotype and phenotype [65]. While GWAS has been successfully applied in human populations for detection of risk genetic variants associated with diseases, when is applied to microorganism populations several confounding factors must be undertaken, such as selective sweeps, recombination frequency, horizontal gene transfer and clonal expansion [97].

In yeast, GWAS has been seldom utilized due to the necessity to genotype a large number of strains from diverse ecological niches, making the QTL mapping the preferred tool for the analysis of complex traits [65]. As far as we know, there is no study focused on nitrogen-related genes using GWAS. However, the recently sequenced “1011 yeasts population” overcome this problem, allowing to apply GWAS on this yeast population and becoming in a powerful resource for a direct association between genotype and phenotype [59]. Thus, we have now the chance to use GWAS as a tool for the identification of genes affecting phenotypes related to wine fermentation, particularly nitrogen utilization, as well as to TORC1 activation.

The GWAS experimental approach has also the advantage to allow the direct phenotypic and genotypic comparison between wild and domesticated yeast strains. *S. cerevisiae* is one of the most important domesticated species, due to its use for food (e.g. bread) and beverage (e.g. beer and wine) fermentations for thousands of years [98], but wild yeast strains also exist in non-human environments, such as tree barks and flowers [49]. However, little is known about the phenotypic effects linked to domestication in yeast, for example, over low nitrogen adaptation.

In this context, it will be of great importance for the yeast researcher community to study the effect that domestication process has had over these and other traits. Given the actual evidence pointing towards the different evolutionary trajectories of wild and domesticated yeast strains [47, 48, 59, 64], we can hypothesize that wild strains of *S. cerevisiae* are better adapted to low nitrogen conditions, being a reservoir of useful alleles to improve industrial yeasts for wine production. Moreover, this domestication process may have caused differences in TORC1 activation between wild and domesticated strains, with some works suggesting the existence of lineage-specific functional divergence for TORC1-associated phenotypes [64, 84, 99, 100], which in turn may have impacted on their fermentative capacities. The disentangling of the genetic bases of yeast adaptation to low nitrogen environments in wine fermentation will help to answer this and other questions, and to take the accumulated knowledge for industrial application in wine industry.

## Conclusions

The study of the yeast natural diversity related to low nitrogen adaptation through QTL mapping and other experimental approaches (e.g. ASE) has helped to better understand the involvement of different genes (and alleles in specific) in phenotypes like growth kinetics, nitrogen sources consumption and fermentation kinetics. Moreover, several studies have left in evidence the central role of the TORC1 signalling pathway in nitrogen utilization and positioned wild yeast strains as a reservoir of useful alleles with potential industrial applications. More studies focused in disentangling the genetic bases of yeast adaptation to low nitrogen environments in wine fermentation will be of great importance to determine the phenotypic effects linked to domestication in yeast over low nitrogen adaptation, as well as to definitely proof that wild strains of *S. cerevisiae* are better adapted to low nitrogen conditions and use their beneficial alleles to improve industrial yeasts for wine production.

## Data Availability

Not applicable.

## Abbreviations

ASB: allele-specific transcription factor binding
ASE: allele specific expression
BSA: bulk segregant analysis
BSR-seq: bulk segregant RNA-seq
GWAS: Genome wide association study
ISA: individual segregant analysis
MHA: massive hemizygote analysis
QTL: quantitative trait locus
WYDC: wine yeast deletion collection
